# Validity and reliability of the Korean version of the gender-friendliness barriers in nursing programs scale

**DOI:** 10.3389/fpsyg.2023.1223368

**Published:** 2023-09-14

**Authors:** Seon-Min Park, Jung-Hee Kim

**Affiliations:** College of Nursing, The Catholic University of Korea, Seoul, Republic of Korea

**Keywords:** validity, reliability, psychological, male, nursing, students

## Abstract

**Introduction:**

The gender-friendliness barriers in nursing programs (GFB-NP) were used to measure perceived gender affinity among male nursing students in nursing education programs. Originally developed in Taiwan, this scale has not been used in Korea. The purpose of this study is to confirm the reliability and validity of the GFB-NP scale for Korean male nursing students.

**Methods:**

A convenience sample of male nursing students enrolled in the 1st to 4th year of nursing departments at five four-year universities located in three cities in Korea was used in the study. To confirm the validity and factor structure of the scale, both exploratory factor analysis and confirmatory factor analysis were employed.

**Results:**

The results support a four-factor structure: Professional acquisition opportunity, peer interaction, sociocultural prejudice, and gender role attitude. We confirmed that the Korean version of the GFB-NP is an appropriate tool for measuring the gender-friendliness educational environment perceived by male nursing students in nursing education.

**Discussion:**

The GFB-NP will serve as a framework for developing counseling and management strategies to help male nursing students successfully adapt to school life within the nursing education curriculum. Research with a longitudinal study design is recommended to investigate the progression of school adaptation through undergraduate program courses.

## Introduction

Globally, nursing is traditionally perceived as a female-dominated profession (Younas et al., 2019). However, recent shifts in societal attitudes toward the nursing profession and increased recognition of nursing expertise have led to an increase in the number of male nurses (Twomey and Meadus, 2016; Lee et al., 2021). As of 2022, male nurses represent over 5% of all licensed nurses in Korea (Korean Nurse Association, 2022) in 2022. In the United States, more than 12% of registered nurses in 2019 are male, demonstrating a significant increase in their representation within the profession (U.S. Bureau of Labor Statistics, 2021). Furthermore, male students constituted 22.4% of the nursing student population in Korea in 2019, totaling 24,058 students (Korean Nurse Association, 2022), indicating that a rapid increase in the number of male nurses can be expected.

Despite the growing number of male nursing students, they often face challenges and unequal opportunities in clinical practicum due to gender discrimination in situations requiring physical contact, such as urinary catheterization, enemas, skin assessments, changing patients’ clothes, and entering rooms exclusively occupied by young female patients (Whiteside and Butcher, 2015; Kim et al., 2016; Zhang and Liu, 2016). Park and Ryeong (2020) highlighted the difficulties of male nursing students in communication stemming from challenges in forming relationships with female students in predominantly female environments. Male nursing students consistently report difficulties adapting to college life because of the lack of a gender-friendly environment (Buthelezi et al., 2015; Powers et al., 2018; Ahn, 2019; Hung et al., 2019). These include negative experiences with support systems owing to the lack of experience and lack of role models of male nurses and male faculty in academic and clinical settings (Kang and Kim, 2015; McKenna et al., 2016; Younas et al., 2019).

A gender-friendly educational environment is one that tries to overcome barriers for nursing students to produce in them a sense of belonging in nursing education programs and clinical practice and provide role models to improve male nursing students’ (a) satisfaction with their major, (b) adaptability to college life, and (c) job adaptability after graduation (Hung et al., 2019). Studies have identified the educational environment as a factor that increases major satisfaction and job adaptability in nursing students (Fang et al., 2018). It has been confirmed that male nursing students develop self-efficacy in a gender-friendly educational environment and that such an environment can influence their long-term career decisions (Dos Santos, 2022).

Male nursing students’ adaptation is influenced by a complex interplay of factors related to educational environments, such as major satisfaction, lack of belonging, interpersonal relationships, faculty-student interactions, gender stereotypes, lack of male faculty and nurse role models, and female-oriented curricula (Lee et al., 2018; Lim and Park, 2018; Powers et al., 2018; Hung et al., 2019). A previous study confirmed that occupational behavior improved as major satisfaction and sense of belonging increased, emphasizing the importance of the educational curriculum and an environment of educational support that need to be created by educators and education decision-makers (Fattahi-Bafghi and Barkhordari-Sharifabad, 2020).

In this way, to enhance the male nursing students’ adaptation to college life, satisfaction with their major, and their sense of belonging, evaluating the gender-friendly educational environment and establishing an appropriate environment becomes critical. To create a gender-friendly educational environment, it is necessary to identify the factors that undergraduate nursing students perceive as barriers to nursing education and develop a reliable and valid instrument for assessment. However, only a few studies have measured the gender-friendly educational environment of male nursing students in Korea. Most of these studies identify gender role conflict (Lee and XiangLian, 2019; Lee and Kim, 2020) and gender stereotypes (Lee and Park, 2018; Han, 2020), with some aiming to evaluate the degree of gender equality in nursing education targeting male and female nursing students (Cho et al., 2022).

In Taiwan, the GFB-NP consists of a total of 20 questions using a 5-point scale to evaluate the gender-equal educational environment recognized by male nursing students. It helps identify the characteristics of male nursing students and fosters an educational environment, including appropriate teaching methods (Hung et al., 2019). The validity and reliability of this tool were verified by measuring the gender-friendly educational environment of male nursing students (Pai et al., 2019).

Therefore, the purpose of this study is to confirm the reliability and validity of the GFB-NP scale for Korean male nursing students, considering culturally different contexts as a measurement tool for evaluating a gender-friendly environment (Hung et al., 2019).

## Methods

### Research design

**Figure 1:**
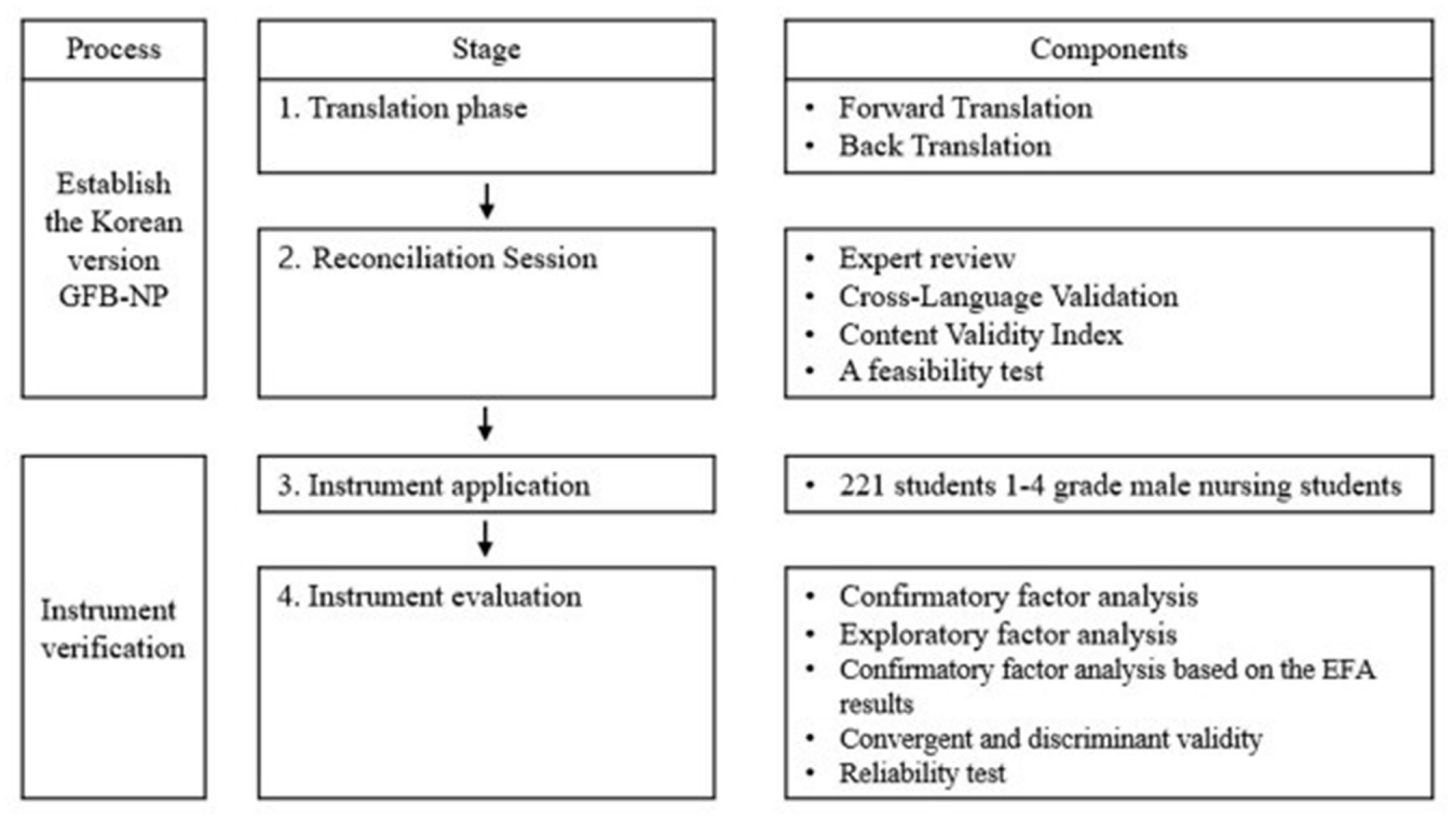
Flow chart the scale development and establishing.

This is a methodological study in which secondary analysis is used to verify the validity and reliability of the GFB-NP scale for male nursing students in Korea. The investigation was conducted in two stages. First, the scale was translated and a feasibility test was performed to establish the scale. In the second phase, data from a convenience sample of male undergraduate nursing students were used to explore and validate the scale’s underlying structure (Figure 1).

### Procedures

#### Phase 1. Establishing the Korean version GFB-NP

The original Chinese version of the GFB-NP was translated into Korean by the first translator, who holds a master’s degree in education, and two bilinguals in both Korean and Chinese. Subsequently, the back translation into Chinese was performed by a second translator, who was fluent in Korean and Chinese. Throughout the reverse translation process, independent work was maintained between the forward and back translators. Finally, the researchers and a back translator discussed and corrected discrepancies between the reverse-translated tool and the original tool, as well as any expressions due to cultural differences and distortion of meaning.

We utilized the item-level content validity index (I-CVI) (Polit et al., 2007) to evaluate whether the Korean version GFB-NP reflected the meaning of the GFB-NP’ items appropriately, which allowed us to validate our version of the scale. For content validity testing of our scale, a panel of five experts reviewed the questionnaire. The external experts included one nursing professor, two clinical practice instructors, and university hospital nurses with more than 10 years of experience. The content validity index for each scale item was calculated using a four-point Likert scale where 1 = not relevant and 4 = highly relevant, indicating the extent to which each item’s content properly identified the factors that undergraduate nursing students perceived as barriers to nursing education. Each item’s I-CVI was calculated as the proportion of experts who rated the item as either quite relevant or very relevant. If the I-CVI value of an item was >0.8 (Polit et al., 2007), the item was deemed valid. Following content validation by the panel of five experts, a 20-item scale was finalized with a content validity index of 0.83 or higher (Polit et al., 2007; Diniz et al., 2019).

Next, a feasibility test using a scale was conducted with four male nursing students in the 2nd and 3rd years of S University. The nursing students demonstrated a high understanding of scale items, and no items were reported as difficult to understand or ambiguously expressed. Based on the result of the feasibility test, no changes were made to the scale. The feasibility test was deemed satisfactory, and the items proceeded to the instrument verification phase.

#### Phase 2. Instrument verification

##### Measure

The GFB-NP (Hung et al., 2019), developed to measure gender friendliness perceptions among male nursing students in nursing education programs, includes six items on Barriers to Belonging, eight items on Barriers to Clinical Practice, and six items on Course-related Barriers. Each item is scored on a 5-point Likert scale ranging from 1 (not at all) to 5 (very much so), with higher scores indicating that male students perceive a more familiar educational environment regarding the three factors. The reliability of the scale, measured by Cronbach’s α, was 0.78 (Hung et al., 2019).

The general characteristics of the participants were age, grade, economic level, living arrangement, religion, academic level, major satisfaction, and conflict experience. These characteristics were included in the questionnaire.

The question regarding major satisfaction was “How satisfied are you with your major in Nursing?” and rated on a satisfaction scale of “very dissatisfied,” “dissatisfied,” “neutral,” and “satisfied or very satisfied.” The question about Conflicts Experience in school life was “Have you ever experienced conflicts in your school life (friends, professors, seniors, and juniors)?” and rated on a scale from “never,” “sometimes” and “frequently.”

### Data collection

This study was approved by the Institutional Review Board of the Catholic University (MC19QESI0115). The data collection occurred between October 29, 2019 to November 22, 2019. A convenience sample of male nursing students in the 1st to 4th year of nursing departments at five 4-year universities was selected. A total of 230 male nursing students participated in the paper-and-pencil survey. Of the 230 distributed questionnaires, 221 were used for the final analysis after excluding nine with insufficient responses (Park and Kim, 2020). All participants had experienced at least one semester of college life after admission. Participants were recruited through recruitment notices posted at universities after obtaining approval from the institution’s managers. They voluntarily agreed to participate in the study after understanding its purpose (Park and Kim, 2020).

The sample size for exploratory factor analysis (EFA) required a ratio of 1:10 for the number of items and participants (Nunnally, 1978), while for confirmatory factor analysis, a minimum of 200 participants was appropriate (Anderson and Gerbing, 1988). The tool, composed of three domains and 20 items, meets these criteria.

### Statistical analysis

The collected data were analyzed using SPSS/WIN (version 18.0) and AMOS (version 22.0 programs, IBM Corp., Armonk, NY, United States). The general characteristics of the participants were analyzed using frequencies, percentages, means, and standard deviations. Construct validity was assessed to determine the tool validity. A confirmatory factor analysis was conducted to evaluate the construct validity of the tool. After ensuring that there were no missing values for any items, the maximum likelihood method was used for estimation, and the model fit was assessed using fit indices. However, as the model fit was low, an EFA was performed. For EFA, Principal component analysis (PCA) was performed, and a scree plot was generated to aid in identifying the number of factors to extract. Factors with an Eigenvalue >1 in the scree plot were considered for extraction. Kaiser–Meyer–Olkin (KMO) and Bartlett’s tests of sphericity were conducted to determine the suitability of the collected data for factor analysis. KMO is a measure of the correlation between variables, with values closer to 1 indicating a better fit. Bartlett’s test of sphericity tests the null hypothesis that the correlation matrix of variables is an identity matrix, verifying whether the diagonal is 1 and the rest are 0. Factor analysis is generally considered suitable if the KMO value is greater than 0.60 and the value of p for Bartlett’s test of sphericity is less than 0.05 (Hair et al., 2013).

Confirmatory factor analysis using the structural equation model was conducted again based on the results of the EFA. In the study, the fit of the model was evaluated using the Root Mean Square Error of Approximation (RMSEA), Comparative Fit Index (CFI), Tucker-Lewis Index (TLI), Normed Fit Index (NFI), and the Akaike Information Criterion (AIC) for the Adjusted Goodness of Fit Index (AGFI). RMSEA values less than 0.05, 0.06–0.08, 0.08–0.10, and greater than 0.1 indicate good, reasonable, mediocre, and poor fit, respectively. CFI, TLI, CFI of 0.90 or higher, and a lower ACI indicate a better fit (Hair et al., 2013).

The average variance extracted (AVE) and construct reliability (CR) were used to analyze the convergent validity of the items. Discriminant validity checked the respective correlation of √AVE scores with other factors (Fornell and Larcker, 1981; Malhotra and Dash, 2015; Hair et al., 2021). The reliability was assessed using Cronbach’s α, a measure of the internal consistency of the tool’s items. Furthermore, we also tested known group comparisons using a t-test and an analysis of variance (ANOVA). The tool’s internal consistency reliability was verified by calculating Cronbach’s α value.

## Results

### General characteristics of the participants

The general characteristics of the participants are presented in Table 1. The participants were 56 first-year students (25.3%), 64 s-year students (29.0%), 65 third-year students (29.4%), and 36 fourth-year students (16.3%), with the average age of the sample being 22.7 years. Academic performance was high in 39 students (17.6%), medium in 142 (64.3%), and low in 40 (18.1%). Regarding major satisfaction, 115 reported being “satisfied” (52.0%), 85 were “neutral” (38.5%), and 21 were “unsatisfied” (9.5%). Interpersonal conflict was reported as “none” by 108 students (48.9%), “occasionally” by 103 students (46.6%), and “frequent” by 10 students (4.5%).

**Table 1:** Fit index of the Korean version GFB-NP (*N* = 221).

Fitness index	ꭓ^2^ (*p*)	ꭓ^2^/df	RMSEA	CFI	NFI	TLI	AIC
Criteria (3 factors)	617.171(0.000)	3.696	0.111	0.685	0.619	0.642	703.17
Korean version GFB-NP (4 factors)	133.964(<0.001)	1.634	0.054	0.942	0.866	0.926	209.96

### Confirmatory factor analysis with 20 items

The results of confirmatory factor analysis based on 20 items of three factors of the original tool were as follows, ꭓ^2^ was 617.17, RMSEA was 0.11, NFI was 0.62, CFI was 0.69, TLI was 0.64, and the fit index was low (Table 2).

**Table 2:** Exploratory factor analysis of the Korean version of GFB-NP (*N* = 221).

Items	Factor			
	1	2	3	4
16. Nursing has traditionally been considered a female occupation, and plans to guide the learning of male nursing students are lacking.	0.80	0.07	0.11	0.19
17. In clinical cases, curriculum content is lacking on the differences in the way men and women communicate.	0.75	0.10	0.20	0.05
18. Suitable guidelines for male nurses are lacking on expressing care or when there is a need for physical contact in their clinical work with female patients.	0.68	−0.04	0.17	−0.03
19. Education on how to provide adequate therapeutic contact is lacking.	0.66	0.20	0.29	−0.01
20. Curriculum content related to the male nurse role model in nursing history is lacking.	0.58	0.15	0.04	0.13
1. There are lesser number of male students in nursing, affecting my sense of belonging.	−0.06	0.81	0.21	0.06
2. There are more female students in nursing, making me feel isolated.	0.31	0.77	−0.06	0.14
3. I do not have a chance to collaborate with other male colleagues.	0.03	0.70	0.36	0.01
4. I do not get opportunities to cooperate with other medical-related colleagues.	0.36	0.60	−0.13	0.26
7. Some clinical units restrict male student participation.	0.13	0.07	0.79	0.10
8. Even among male patients, preference is for receiving care from female nurses.	0.26	0.19	0.67	0.07
9. In female patients, preference is to receive care from female nurses.	0.27	0.03	0.48	0.20
10. Gender differences sometimes come between me and my patient when providing care and attention.	0.09	0.01	0.27	0.80
11. I feel embarrassed when explaining matters involving sensitive topics to female patients.	0.18	0.07	0.35	0.73
12. In delivering professional services involving close-proximity care and attention, female nurses are often more suitable to the task than male nurses.	0.01	0.29	−0.18	0.55
Engine values	4.45	1.75	1.39	1.15
Explained variance (%)	29.65	11.65	9.25	7.65
Cumulative variance (%)	29.65	41.30	50.55	58.20

Factor 1, professional acquisitions opportunity; Factor 2, peer interaction; Factor 3, sociocultural prejudice; Factor 4, gender role attitude.

### EFA with 20 items

Since respondents’ reactions to and structures of measurement tools may vary due to the characteristics and cultural differences of the study population and situation, an EFA was conducted to create a model or structure of the tool (Pett et al., 2003). The 20-item tool used in this study revealed six factors, with some subfactors having only two corresponding items, low commonality, or other unsuitable items. Items that were conceptually unrelated and items with high loadings on multiple factors were excluded while selecting items that met the conditions.

The minimum recommended value for factor loadings is 0.3 (Merenda, 1997). Items with factor loadings below 0.3 and lacking conceptual relevance, such as No. 5, “There was no opportunity to cooperate with other male students” (0.205) and No. 6, “There was no opportunity to cooperate with other health care peers” (0.210), were excluded. No. 14, “Male students will face different conditions and restrictions than what their female counterparts do during Obstetrics and Gynecology practice.” was excluded as an item with simultaneously high loadings for multiple factors. By additionally comparing the number of factors and items while checking the fit indices to determine if the overall model fit improved, item No. 13, “In delivering professional services involving support and encouragement to the patients, female nurses are often more suitable to the task than male nurses.” and No. 15, “Nursing is originally a female occupation, and the nursing curriculum design does not adequately prepare male nursing students.” were excluded.

**Figure 2:**
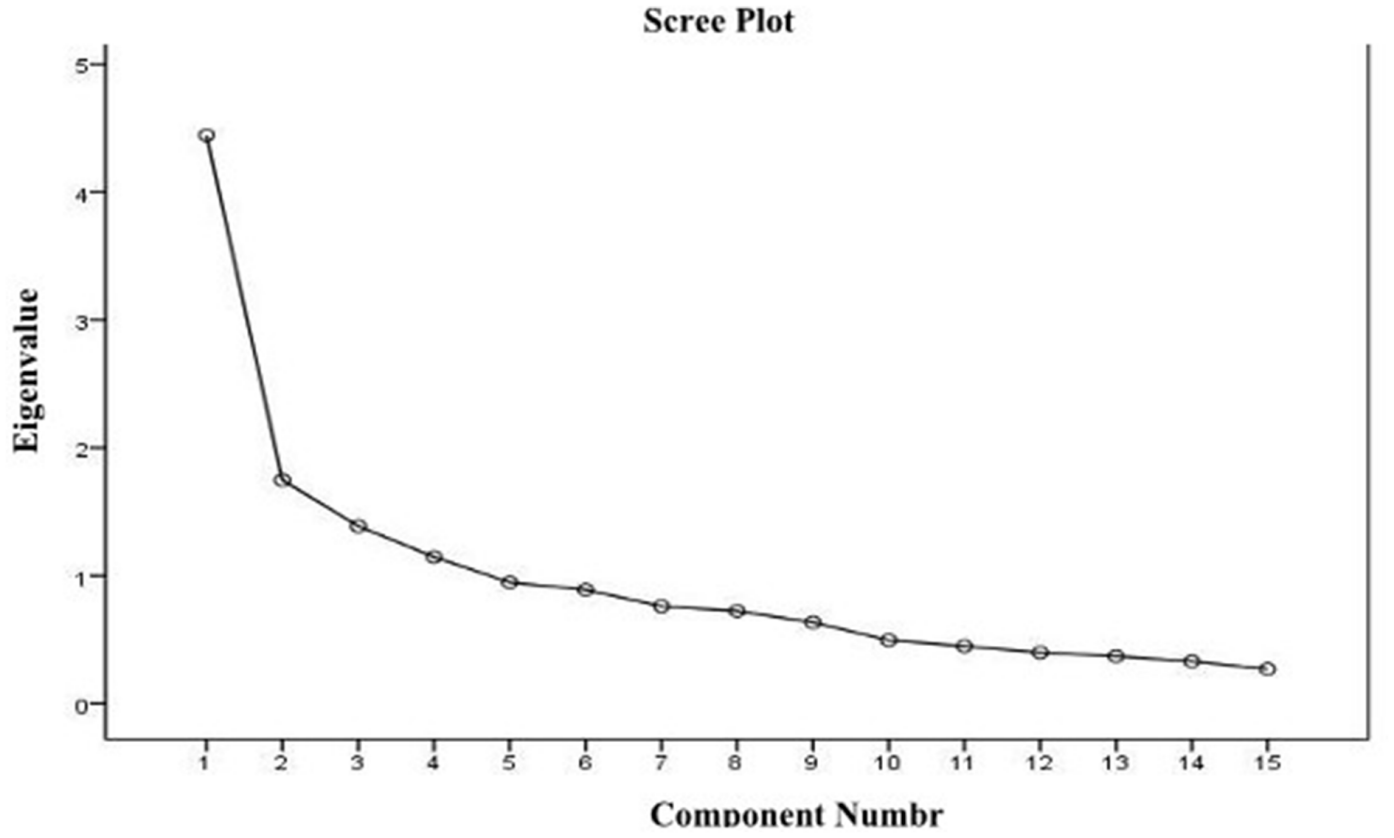
The scree plot obtained exploratory factor analysis.

After conducting factor analysis using the final 15-item tool, four factors (eigenvalue >1) were extracted. Moreover, the scree plot indicated that the 4-factor structure was suitable for the scale (Figure 2). Factor loadings ranged from a minimum of 0.48 to a maximum of 0.80 for each factor, with the first factor explaining 19.08%, the second factor explaining 15.25%, the third factor explaining 12.75%, and the fourth factor explaining 11.15%. The cumulative variance contribution rate (%) of the four factors was 58.20%. The first factor, with five items, was termed “Professional acquisition opportunity”; the second factor with four items was termed “Peer interaction”; the third factor with three items was termed “Sociocultural prejudice”; and the fourth factor with three items was termed “Gender role attitude” (Table 2).

### Confirmatory factor analysis with 15 items based on the EFA results

Confirmatory factor analysis was conducted to verify whether the 15 items derived from the EFA were appropriately extracted into four factors. The results of the confirmatory factor analysis showed ꭓ^2^ = 194.600, RMSEA = 0.077, CFI = 0.876, NFI = 0.805, TLI = 0.845, and ACI = 266.60. To improve the model fit, the value of ꭓ^2^ was lowered using the corrected index values between the error terms e8 and e9 of the “peer interaction” factor and e1 and e2 of the “professional acquisition opportunity” factor. The results of the modified model fit showed ꭓ^2^ = 133.964 (*p* < 0.001), RMSEA = 0.054, RCFI = 0.942, NFI = 0.866, and TLI = 0.926; the model fit was satisfactory (Table 2).

**Figure 3:**
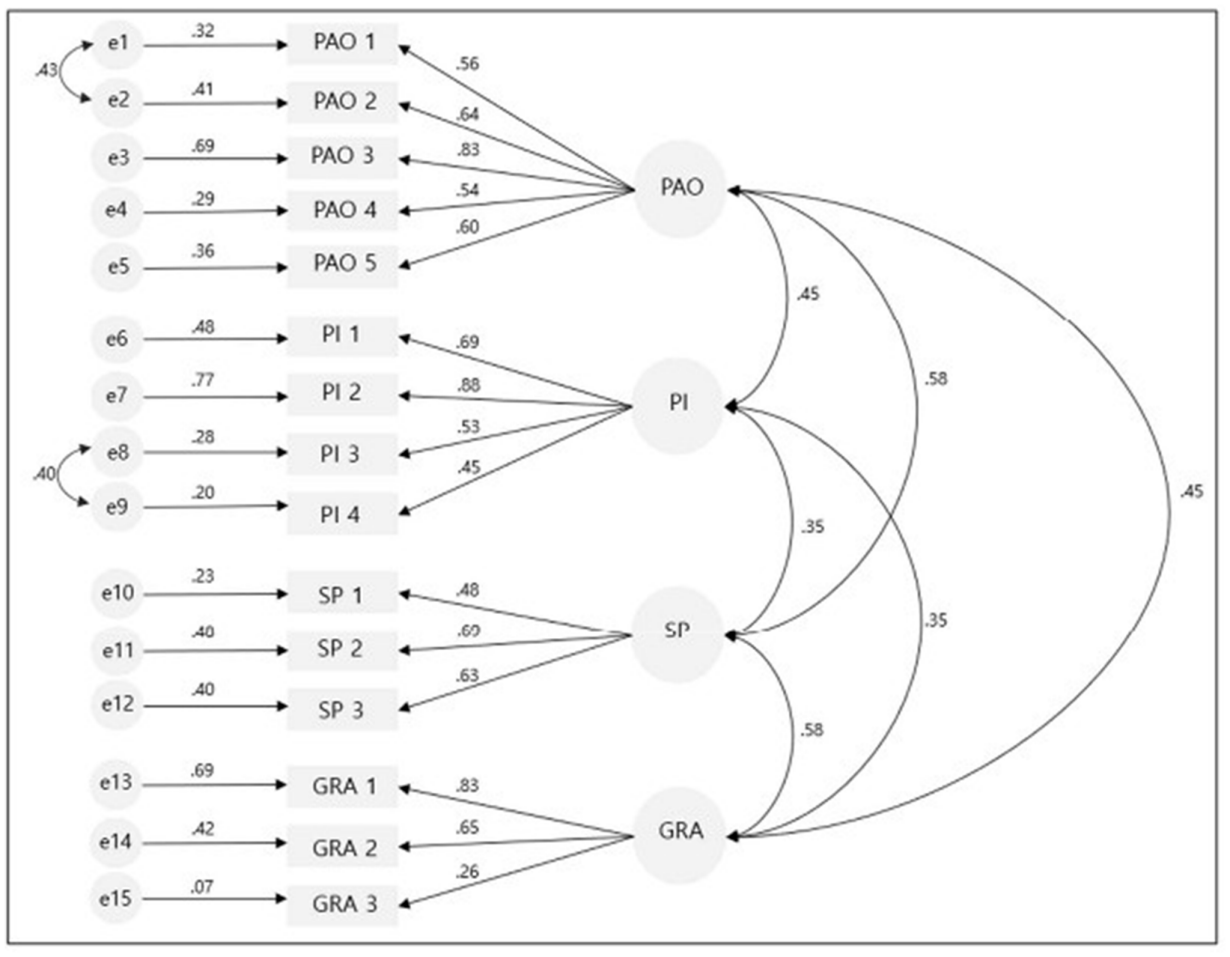
Confirmatory factor analysis of the Korean version GFB-NP. e1–e15, measurement error; PAO, professional acquisitions opportunity; PI, peer interaction; SP, sociocultural prejudice; GRA, gender role attitude.

All paths from the latent variables, “Professional acquisition opportunity,” “Peer interaction,” “Sociocultural prejudice,” and “Gender role attitude,” to the measured variables were significant at the 0.001 significance level (Table 3). Consequently, 15 items across four factors were finally selected for the Korean Version GFB-NP (Figure 3).

**Table 3:** Comparison of according to general characteristics (*N* = 221).

Variables	Categories	GFB-NP	
		Mean ± SD	t/F (*p*)
Age (years)	20 ≤ ^a^ 21 ~ 25^b^ >25^c^	61.70 ± 11.57 57.60 ± 10.39 54.89 ± 10.83	4.76(0.010) a > c
Grade	1st^a^ 2nd^b^ 3rd^c^ 4th^d^	62.16 ± 10.98 58.48 ± 9.99 55.43 ± 11.99 57.83 ± 9.21	3.96(0.009) a > c
Major satisfaction	Satisfied Neutral Dissatisfied	59.08 ± 10.99 59.12 ± 10.46 51.80 ± 11.13	4.33(0.014) a,b > c
School life conflict experience	Never Sometimes Frequently	60.88 ± 10.95 56.70 ± 10.35 49.20 ± 9.95	8.00(0.001) a,b > c

a,b,c,d: Scheffe’s method of multiple comparison.

### Convergent and discriminant validity

Convergent validity can be verified by utilizing the CR and AVE values. In Table 4, which presents the results of the validity analysis, all latent variables have CR values above 0.6 (Bagozzi and Yi, 1988); however, the AVE is lower than 0.5. Generally, CRs are considered meaningful if they are above 0.7 and acceptable if they are between 0.6 and 0.7. Considering that the AVE values do not meet the recommended criterion of 0.5 (Fornell and Larcker, 1981; Malhotra and Dash, 2015), it is suggested that convergent validity should be primarily checked based on CR since AVE is a more conservative measure. Therefore, we can conclude that all the variables have convergent validity.

**Table 4:** Confirmatory factor analysis of the Korean version GFB-NP.

Factors	Item no.	Estimate		S.E	C.R	AVE	Correlation (square of the correlation)			
		B	*β*				PAO	PI	SP	GRA
PAO	16	1.00	0.54		0.778	0.418	1.000			
	17	1.07	0.64	0.12						
	18	1.34	0.85	0.18						
	19	0.92	0.54	0.15						
	20	1.03	0.60	0.16						
PI	1	1.00	0.69		0.685	0.367	0.448 (0.200)	1.000		
	2	1.27	0.88	0.15						
	3	0.74	0.53	0.11						
	4	0.69	0.45	0.12						
SP	7	1.01	0.49	0.19	0.604	0.341	0.583 (0.340)	0.346 (0.120)	1.000	
	8	1.27	0.70	0.19						
	9	1.00	0.61							
GRA	10	3.66	0.84	1.14	0.617	0.385	0.451 (0.203)	0.347 (0.120)	0.580 (0.336)	1.000
	11	2.85	0.64	0.88						
	12	1.00	0.25							

GFB-NP, gender-friendliness barriers in nursing program; B, unstandardized regression weights; β, unstandardized regression weights; SE, standard error; CR, critical ratio; PAO, professional acquisitions opportunity; PI, peer interaction; SP, sociocultural prejudice; GRA, gender role attitude.

Discriminant validity is obtained when the AVE exceeds the multiplicative value of the correlation coefficient between concepts. According to Table 4, when comparing the AVE with the magnitude of the multiplicative value of the correlation coefficient between all latent variables, the AVE value of all factors was larger, thereby securing discriminant validity.

### Known group test

Significant differences were observed in the Korean version GFB-NP based on age, grade, major satisfaction, and school-life conflict experience. The scores of the Korean version GFB-NP were higher in the first year than in the third year (*F* = 3.96, *p* = 0.009) and significantly higher in the group with high major satisfaction (*F* = 14.33, *p* < 0.001) compared to other groups with lower satisfaction or dissatisfaction. The Korean version GFB-NP was significantly related to the experience of conflict in school life (*F* = 8.00, *p* = 0.001) (Table 3).

### Reliability

The overall reliability of the 15-item Korean version GFB-NP was 0.82, which is considered satisfactory. The reliability for each subdomain was as follows: for “Professional acquisition opportunity,” 0.78; “Peer interaction,” 0.76; “Sociocultural prejudice,” 0.61; and “Gender role attitude,” 0.60 (Table 5).

**Table 5:** Reliability according to the four factors of the GFB-NP (*N* = 221).

Factors	Number of items	Cronbach’s α
Professional acquisitions opportunity	5	0.78
Peer interaction	4	0.76
Sociocultural prejudice	3	0.61
Gender role attitude	3	0.60
GFB-NP	15	0.82

## Discussion

This study was conducted to verify the validity and reliability of the GFB-NP scale developed to measure gender affinity as perceived by male nursing students in a nursing education program. We tested the construct’s validity using EFA and Confirmatory factor analysis. The goodness of fit of the 20-item 3-factor model (GFB-NP) was low and did not reach the mood, but the goodness-of-fit index of the 15-item 4-factor model (the Korean Version GFB-NP) met the criterion. Although no previous studies have compared factor structures, it is likely that cultural differences exist and can affect the factor structure. Our study results highlight the need to explore cultural differences when using heterogeneous cultural tools. The result showed that while the original GFB-scale NP (Hung et al., 2019) consisted of three factors, “Belongingness barrier,” “Clinical practice barrier,” and “Curriculum barrier,” in the Korean version GFB-NP, “Curriculum barrier” and “Belongingness barrier” items were renamed as “Professional acquisition opportunity” and “Peer interaction,” respectively. The “Clinical practice barrier” factor was divided into “Sociocultural prejudice” and “Gender role attitude” and renamed accordingly.

In this study, Factor 1, “Professional acquisition opportunity,” refers to the improvement of professional identity; decrease in turnover intention; enhancement of nursing satisfaction; promotion of health recovery and well-being; improvement of nursing quality; and acquisition of social recognition through nursing education and experience, research activities, personal qualities, and self-directed training over an adequate period (Lee and Kim, 2019). One item was removed from the original 6-item “Curriculum barriers” factor, resulting in five items related to barriers, such as learning guidance plans and guidelines for male students in the nursing education curriculum and lack of role models. The item “Nursing is originally a female occupation, and the nursing curriculum design does not adequately prepare male nursing students” was removed from the “Curriculum barriers” factor. This difference in item composition may be due to the fact that Hung et al. (2019) did not include male nursing students without clinical practice experience, while this study targeted all male nursing students currently enrolled in nursing schools. According to previous studies, the less clinical experience one has, the more gender-friendly their perception about the nursing education environment is (Park and Kim, 2020). Once clinical practice education begins, they experience stress and anxiety from the hospital environment and face difficulty taking appropriate actions in various unpredictable nursing situations (Kim and Song, 2020).

Factor 2, “peer interaction,” consisted of items that indicated the degree of belongingness barriers through interactions with nursing- and medical-related peers. Two items were removed from the original 6-item “Belongingness Barriers” factor: “There was no opportunity to cooperate with other male students” and “There was no opportunity to cooperate with other health care peers.” In Taiwan, where the original tool was developed, male nurses accounted for 3% of all licensed nurses (Taiwan Union of Nurses Association, 2020), whereas in Korea, male nurses accounted for 4.3% (Korean Nurse Association, 2020), and their numbers are increasing. Relatively more male nursing students have experience working with male nurses or accepting them as role models (Younas and Sundus, 2018a,b; Kim and Song, 2020), which might explain why the two items removed from Factor 2 were deemed non-functional in Korea.

Factors 3 and 4 were originally a single factor with eight items called “clinical practice barriers.” However, in this study, two items were removed: “In delivering professional services involving support and encouragement to the patients, female nurses are often more suitable to the task than male nurses.” and “Male students will face different conditions and restrictions than what their female counterparts do during Obstetrics and Gynecology practice.” Consequently, the factors were divided into “sociocultural prejudice” and “Gender role attitude,” with each consisting of 3 items related to barriers caused by prejudice from others and barriers related to gender role attitudes perceived by oneself. This indicates that while there may be difficulties in providing nursing services to female patients, such as showing meticulous and warm care and explaining sensitive content (Whiteside and Butcher, 2015; Park and Kim, 2020) men who choose a female-oriented field tend to have lower gender stereotypes than men who choose other majors (Lee and Park, 2018). The Korean version of the GFB-NP scale reflects the changing gender stereotypes among male nursing students.

In terms of age, individuals under the age of 20, compared to those over 25, and students in the first grade, compared to those in the third grade were more likely to perceive a gender-friendly educational environment. This result is consistent with previous studies that indicate that the older the age and the higher the grade, the more negatively experienced female-centered curriculum and clinical practice (Powers et al., 2018; Ahn, 2019; Hung et al., 2019). The degree to which the nursing education process was perceived as a gender-friendly educational environment was significantly higher among the group with high major satisfaction and low experience of interpersonal conflict. Therefore, to increase the gender-friendly educational environment recognized by male nursing students, the development and implementation of an educational program that considers the grade level is necessary.

To verify the reliability of the Korean version of the GFB-NP, the internal consistency was examined, and the overall reliability Cronbach’s α value was 0.82. The factors were as follows: factor 1 = 0.78, factor 2 = 0.76, factor 3 = 0.61, and factor 4 = 0.60. When considering the factor structure, α values exceeding 0.7 were discovered for two factors, whereas factor consisting of 3 items had a lower but acceptable Cronbach’s α (0.61, 0.60). As Cronbach’s α is very sensitive to the number of items in scales, it is common to detect lower α values in factors with a few items (Streiner, 2003). For newly modeled tools, a Cronbach’s α value of 0.70 or higher establishes internal consistency reliability (Iacobucci and Duhachek, 2003), confirming the reliability of the tool for use among male nursing students.

Based on the study finding, it is essential to assess and evaluate the level of gender-friendly educational environment among male nursing students in educational practice. This scale can be applied to male nursing students of all grades, proving a valid and reliable instrument for measuring the gender-friendly educational environment of nursing undergraduates. The present study will assist nurse educators in developing a gender-friendly nursing curriculum designed to enhance the level of male nursing students’ adaptation to school life while accommodating their characteristics. The GFB-NP will serve as a framework for developing counseling and management strategies to help male nursing students successfully adapt to school life within the nursing education curriculum. Research with a longitudinal study design is recommended to investigate the progression of school adaptation through undergraduate program courses. Ultimately, this research may contribute to improving male nursing students’ adjustment to university life, their satisfaction with their major, and forming a positive career outlook.

However, this study had several limitations. Firstly, since the participants responded with a self-reporting questionnaire, biased perceptions and capabilities for desirable answers may have affected the validity of the results. The data used convenience sampling, which also limited data interpretation. Second, this study collected data from students who had not experienced clinical practicum. It is possible that perceptions of gender-equitable educational environments may change depending on clinical practice experience, potentially affecting the validity and reliability of the tool. Therefore, future studies should consider measuring the tool separately by grade level and clinical practice experience of the study participants. Thirdly, since test–retest reliability was not assessed as part of the reliability test, a limitation may exist regarding the difficulty to secure the stability of test scores. Nevertheless, this study’s scale could overcome the limitations of existing scales developed from different cultures by verifying the validity and reliability of the GFB-NP scale, tailored to measure gender affinity as perceived by male nursing students in a nursing education program in Korea.

## Data availability statement

The raw data supporting the conclusions of this article will be made available by the authors, without undue reservation.

## Ethics statement

The studies involving humans were approved by the Institutional Review Board of the Catholic University. The studies were conducted in accordance with the local legislation and institutional requirements. The participants provided their written informed consent to participate in this study. Written informed consent was obtained from the individual(s) for the publication of any potentially identifiable images or data included in this article.

## Author contributions

SM and JH: conceptualization. SM: formal analysis, and visualization. SM and JH: methodology. All authors read and approved the final manuscript.

## Conflict of interest

The authors declare that the research was conducted in the absence of any commercial or financial relationships that could be construed as a potential conflict of interest.

## Publisher’s note

All claims expressed in this article are solely those of the authors and do not necessarily represent those of their affiliated organizations, or those of the publisher, the editors and the reviewers. Any product that may be evaluated in this article, or claim that may be made by its manufacturer, is not guaranteed or endorsed by the publisher.
